# Adipose‐derived mesenchymal stem cells differentiate into pancreatic cancer‐associated fibroblasts *in vitro*

**DOI:** 10.1002/2211-5463.12976

**Published:** 2020-10-09

**Authors:** Yoshihiro Miyazaki, Tatsuya Oda, Nobuhito Mori, Yasuyuki S. Kida

**Affiliations:** ^1^ Department of Gastrointestinal and Hepato‐Biliary‐Pancreatic Surgery Faculty of Medicine University of Tsukuba Tsukuba Japan; ^2^ Cellular and Molecular Biotechnology Research Institute National Institute of Advanced Industrial Science and Technology (AIST) Tsukuba Japan; ^3^ Advanced Photonics and Biosensing Open Innovation Laboratory The National Institute of Advanced Industrial Science and Technology (AIST) Tsukuba Japan

**Keywords:** adipose, cancer‐associated fibroblast, heterogeneity, mesenchymal stem cell, pancreatic ductal adenocarcinoma, tumor microenvironment

## Abstract

Cancer‐associated fibroblasts (CAFs) are key components of the dense, proliferating stroma observed in pancreatic ductal adenocarcinoma (PDAC), and CAF subpopulations drive tumor heterogeneity and play a major role in PDAC progression and drug resistance. CAFs consist of heterogenous subpopulations such as myoblastic CAF (myCAF) and inflammatory CAF (iCAF), and each has distinct essential roles. However, it is not clear how CAF subpopulations are formed in PDAC. Adipose‐derived MSCs (AD‐MSCs), which possess a high multilineage potential and self‐renewal capacity, are reported to be one of the *in vivo* CAF sources. Here, we aimed to investigate whether AD‐MSCs can act as precursors for CAFs *in vitro*. We recorded morphological features and collected omics data from two *in vitro* co‐culture models for recapitulating clinical PDAC. Additionally, we tested the advantages of the co‐culture model in terms of accurately modeling morphology and CAF heterogeneity. We showed that AD‐MSCs differentiate into two distinct CAF subpopulations: Direct contact co‐culture with PDAC cell line Capan‐1 induced differentiation into myCAFs and iCAFs, while indirect co‐culture induced differentiation into only iCAFs. Using these co‐culture systems, we also identified novel CAF markers that may be helpful for elucidating the mechanisms of CAFs in the tumor microenvironment (TME). In conclusion, AD‐MSCs can differentiate into distinct CAF subtypes depending on the different co‐culture conditions *in vitro*, and the identification of potential CAF markers may aid in future investigations of the mechanisms underlying the role of CAFs in the TME.

## Graphical abstract

Here, we report that adipose‐derived MSC can differentiate into distinct CAF subtypes *in vitro* depending on the co‐culture conditions used; in addition, we have identified potential CAF markers that may aid in investigating the mechanisms underlying the role of CAFs in the tumor microenvironment.

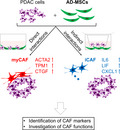

**Abbreviations**
AD‐MSC: adipose‐derived MSC
apCAF: antigen‐presenting CAF
CAF: cancer‐associated fibroblasts
CDX: cell line‐derived xenograft
ECM: extracellular matrix
FACS: fluorescence‐activated cell sorting
GEMM: genetically engineered mouse model
GO: gene ontology
HE: hematoxylin and eosin
iCAF: inflammatory CAF
IF: immunofluorescence
IHC: immunohistochemistry
MSC: mesenchymal stem cell
myCAF: myoblastic CAF
PDAC: pancreatic ductal adenocarcinoma
qPCR: quantitative real‐time polymerase chain reaction
RFP: red fluorescent protein
RNA‐seq: RNA sequencing
TME: tumor microenvironment
αSMA: α‐smooth muscle actin

Pancreatic ductal adenocarcinoma (PDAC) has the worst outcome among all cancers, with a 5‐year survival rate of < 10% [1]. The poor prognosis of PDAC may be explained by its unique histological characteristics, namely stromal desmoplasia, which involves extensive stromal proliferation that constitutes up to 90% of the total tumor mass [2]. Among the various components of the desmoplastic stroma, including fibroblasts, immune cells, vasculature, and extracellular matrix (ECM), cancer‐associated fibroblasts (CAFs) are a major component of the tumor microenvironment (TME) in PDAC, which produce various types of ECM proteins and soluble signaling molecules. Previous studies using *in vivo* murine models such as cell line‐derived xenografts (CDXs), patient‐derived xenografts [3, 4], or genetically engineered mouse models (GEMMs) have reported that CAFs play a major role in facilitating tumor growth by attenuating drug responses as well as immunosurveillance [5, 6]. In contrast, *in vitro* models are advantageous, as they have a relatively low cost and allow pharmacological manipulation, genetic modifications, and imaging analysis. They can be useful in understanding cellular mechanisms such as the interaction between cancer cells and CAFs.

The major sources of CAFs in PDAC are pancreatic stellate cells and bone marrow‐derived mesenchymal stem cells (MSCs) [7, 8]. Adipose‐derived MSCs (AD‐MSCs) are another source of CAFs. They possess a high multilineage potential and self‐renewal capacity. Recent studies have revealed that adipose tissue is a source of CAFs and exerts a tumor‐promoting role in PDAC [9, 10]. The pancreas is a retroperitoneal organ surrounded by adipose tissue. In addition, adipose‐derived stromal cells have a homing capacity and are recruited to experimental tumors in mouse models [11]. Also, secreted factors including exosomes from breast, ovarian, and prostate cancer cells convert them into CAF‐like phenotypes [12, 13, 14]. It is a fair assumption that cells in the adipose tissue would contribute to the formation of the TME in PDAC. Further, previous studies have revealed that MSCs perform a tumor‐promoting role in breast cancer [15, 16], stemness‐ and chemoresistance‐promoting roles in gastric cancer[17], and a metastasis‐promoting role in ovarian cancer [18]. While it is known that AD‐MSCs have the capacity to differentiate into CAFs, the exact mechanism and role of these cells in PDAC remain unclear.

Initially, CAFs were believed to be mainly composed of myofibroblasts characterized by high α‐smooth muscle actin (αSMA) expression [19, 20]. However, recent studies have identified heterogeneous CAF subpopulations such as myoblastic CAFs (myCAFs) and inflammatory CAFs (iCAFs). myCAF is a CAF subpopulation with an elevated expression of αSMA that are located adjacent to cancer cells, whereas iCAFs, which are located further away from cancer cells, exhibit low αSMA expression and are characterized by the secretion of inflammatory mediators such as IL‐6 [21]. Distinguishing between these different populations of cells is important to understand the mechanisms underlying the differentiation of CAF cells.

Herein, we aimed to investigate whether AD‐MSCs can act as progenitors for CAFs *in vitro*. Using co‐culture models for recapitulating clinical PDAC, the morphological features and omics data were obtained from the two *in vitro* co‐culture systems; additionally, the advantages of the co‐culture model in terms of accurately modeling morphology and CAF heterogeneity were tested.

## Materials and methods

### Cells and culture conditions

The human immortalized AD‐MSC cell line ASC52telo (ATCC SCRC‐4000) and human pancreatic cancer cell line Capan‐1 (ATCC HTB‐79), MIAPaCa‐2 (CRL‐1420), and SUIT‐2 (JCRB1094) were utilized in the present study. These cells were maintained in Dulbecco's modified Eagle's medium (DMEM; FUJIFILM Wako Pure Chemical Corp., Osaka, Japan) supplemented with 20% FBS, 1% nonessential amino acids, 1% streptomycin–penicillin at 37 °C in a humidified atmosphere containing 5% CO_2_. AD‐MSCs were labeled with GFP, whereas Capan‐1 cells were labeled with red fluorescent protein (RFP) using lentiviral transduction. After genetic engineering for GFP expression of AD‐MSC, we confirmed that AD‐MSCs did not lose their original characteristics like auto‐differentiation, senescence, or weak stemness (data not shown). To establish clinical CAFs isolated from the patients with PDAC, PDAC tumor sections were minced and 2‐mm tumor pieces were plated onto a gelatin‐treated 3.5‐cm dish in the HFDM‐1 medium (Cell Science & Technology Institute, Sendai, Japan), supplemented with 1% streptomycin–penicillin and 5% FBS. The dishes were then incubated under 5% CO_2_, 20% O_2,_ and at 37 °C. Under these culture conditions, CAFs selectively expanded, while the remaining PDAC cells were depleted after a few passages.

### Patient sample collection

Human pancreatic cancer tissue was obtained with patient written informed consent. This study was approved by the Research Ethics Board of the University of Tsukuba and was carried out in accordance with the Declaration of Helsinki principles. Approval was obtained from the Tsukuba Clinical Research & Development Organization (T‐CReDO protocol number: H25‐119, R01‐193). A total of six resected pancreatic cancer specimens were used in the present study. For the application of these clinical samples for research purposes, a written informed consent was obtained from all patients.

### *In vitro* co‐culture assay

AD‐MSC and Capan‐1 cells were co‐cultured under two different conditions: direct and indirect transwell co‐culture. In the direct co‐culture method, AD‐MSCs (4 × 10^5^ cells) and Capan‐1 cells (4 × 10^5^ cells) were mixed and kept in 6‐well culture plates. For the indirect transwell co‐culture, 4 × 10^5^ AD‐MSCs were seeded in the lower compartment, while 4 × 10^5^ Capan‐1 cells were seeded in the upper compartment of a transwell membrane (Falcon® Permeable Support for 6‐well plates with 3.0‐µm Translucent High‐Density PET Membrane #353092; Corning, Corning, NY, USA). Time‐lapse imaging was performed using the JuLI™ FL Fluorescence Cell History Recorder (NanoEnTek Inc., Seoul, Korea). Images were recorded every 10 min for 7 days.

### Immunofluorescence (IF) staining of cells in monocultures and co‐cultures

The cells were fixed with 100% methanol for 10 min at −20 °C and then washed thrice for 5 min with IF buffer solution (10 × stock: 38.0 g NaCl, 9.38 g Na_2_HPO_4_, 2.07 g NaH_2_PO_4_, 2.5 g NaN_3_, 5.0 g BSA, 10 mL Triton X‐100, and 2.5 mL Tween‐20 in 500‐mL PBS), followed by treatment with a blocking solution (3% BSA in 1 × IF washing solution) for 30 min. The cells were then incubated for 1 h at 25 °C with mouse SMA antibody (1 : 400, ab7817; Abcam, Cambridge, UK), rabbit anti IL‐6 antibody (1 : 200, ab6672; Abcam), or rabbit anti GFP antibody (1 : 400, #598; Medical & Biological Laboratories, Nagoya, Japan) in 1 × IF buffer. After three washing cycles with 1 × IF buffer, the cells were incubated for 1 h with Alexa Fluor 488 Goat anti‐mouse IgG antibody (Invitrogen, Carlsbad, CA, USA), Alexa Fluor 568 Goat anti‐rabbit IgG antibody (Invitrogen), or Alexa Fluor 647 Goat anti‐rabbit IgG antibody (Invitrogen), respectively, diluted 1 : 400 in 1 × IF buffer solution. For counterstaining, the cell nuclei were stained with Hoechst 33342 (Thermo Fisher Scientific, Waltham, MA, USA). After three washing cycles with 1 × IF buffer, the slides were mounted and imaged using a fluorescence microscope [BZ‐710 (Keyence, Osaka, Japan) and ECLIPSE T*i*2 (Nikon, Tokyo, Japan)].

### RNA extraction and quantitative real‐time PCR (qPCR)

Total RNA was prepared using TRI Reagent (Molecular Research Center, Inc., Cincinnati, OH, USA), according to the manufacturer's instructions. Subsequently, 500 ng of total RNA was used to generate cDNA using the RevaTra Ace reverse‐transcription reagents (TOYOBO, Osaka, Japan), according to the manufacturer’s instructions. qPCR was performed using commercially available gene‐specific PrimeTime qPCR probes (listed below; purchased from INTEGRATED DNA TECHNOLOGIES, Coralville, CA, USA) and 2 × Thunderbird Probe qPCR mix (TOYOBO), following the manufacturer's instructions. Gene‐expression levels were normalized to those of glyceraldehyde‐3‐phosphate dehydrogenase (*GAPDH*). The following PrimeTime qPCR probes were used (Hs, Human probes): C‐X‐C motif chemokine ligand 1 (*CXCL1*), Hs.PT.58.39039397; GAPDH, Hs.PT.39a.22214836; actin alpha 2, smooth muscle (*ACTA2*), Hs.PT.56a.2542642; Interleukin 6 (*IL6*), Hs.PT.58.40226675; leukemia inhibitory factor (*LIF*), Hs.PT.58.27705899; connective tissue growth factor (*CTGF*), Hs.PT.58.14485164.g; tropomyosin‐1 (*TPM‐1*), Hs.PT.58.39747432. For the co‐culture samples, RFP‐labeled Capan‐1 and GFP‐labeled AD‐MSC cells were isolated from the co‐culture using fluorescence‐activated cell sorting (FACS)‐Aria III (BD Bioscience, Franklin Lakes, NJ, USA), according to the manufacturer's instructions, and then analyzed.

### RNA sequencing (RNA‐seq)

Total RNA was isolated using the TRI Reagent. The library preparation and sequencing were performed at Macrogen, Japan, with the Truseq library prep kit and NovaSeq 6000 (Illumina, San Diego, CA, USA) to produce 150‐bp paired‐end reads. The acquired data from two independent devices for each condition were mapped and quantified using STAR (2.7.1a) [22] and RSEM [23] (1.3.1) with hg38 as the reference genome and Ensemble GRCh38 as the gene annotation. Subsequently, differentially expressed genes were analyzed using iDEP.91 [24]. Gene ontology (GO) enrichment analysis was performed using the database for annotation, visualization, and integrated discovery (DAVID) [25, 26]. Raw sequences in the FASTQ format were deposited at the DNA Data Bank of Japan (DDBJ, accession number DRA010287).

### Mouse model and *in vivo* experiments

Female nude mice (Balb/c nu/nu), aged 8 weeks, were purchased from Japan CLEA Inc. (Tokyo, Japan) and used in the experiments. In the cell xenograft model, 1 × 10^6^ pancreatic cancer cells were subcutaneously transplanted into the mice. After 4 weeks, the mice were sacrificed, and all subcutaneous tumors were excised. Subsequently, mouse tumor tissues were fixed in 10% formalin neutral‐buffered solution, embedded in paraffin, and cut into 2‐µm‐thick sections. Hematoxylin and eosin (HE) staining was performed according to the standard protocol. All mouse experiments were approved by the Institutional Animal Care and Use Committee of the respective institutes of National Institute of Advanced Industrial Science and Technology (AIST, 2020‐310) and the Ethics Committee of the University of Tsukuba (19‐028).

### Immunohistochemical staining of the clinical PDAC tissues

All staining procedures were performed on 3‐µm‐thick sections of human tissues. For immunohistochemistry (IHC), the sections were deparaffinized before performing antigen retrieval at 121 °C in an autoclave for 10 min in 10 mm sodium citrate buffer (pH 6.0). Endogenous peroxidases were blocked by treating the sections with 3% H_2_O_2_ solution (Envision Plus System; Dako, Santa Clara, CA, USA). The primary antibodies used for IHC were as follows: αSMA (1 : 400, ab5694; Abcam), LIF (1 : 500, ab113262; Abcam), SEMA7A (1 : 100, HPA042273; Atlas Antibodies, Stockholm, Sweden), and HAS1 (1 : 200, GTX82799; GeneTex, Irvine, CA, USA). The labeled antigens were visualized using the chromogen 3,3′‐diaminobenzidine tetrahydrochloride; hematoxylin was used as a nuclear counterstain. The slides were imaged using a fluorescence microscope (BZ‐710; Keyence).

### Statistical analysis

Data were represented as the mean ± SD. The differences between each group were compared using the unpaired two‐tailed Student's *t*‐test or one‐way analysis of variance (ANOVA) with Tukey's method for multiple comparison tests. All data were evaluated using ANOVA in microsoft office excel (Microsoft, Redmond, WA, USA) or the statistical analysis software package spss version 25.0 (IBM SPSS Statistics, Armonk, NY, USA). *P* < 0.05 was considered statistically significant. The error bars in the figures represent the SD.

## Results

### Capan‐1‐induced MSC differentiation into CAFs

**Fig. 1:**
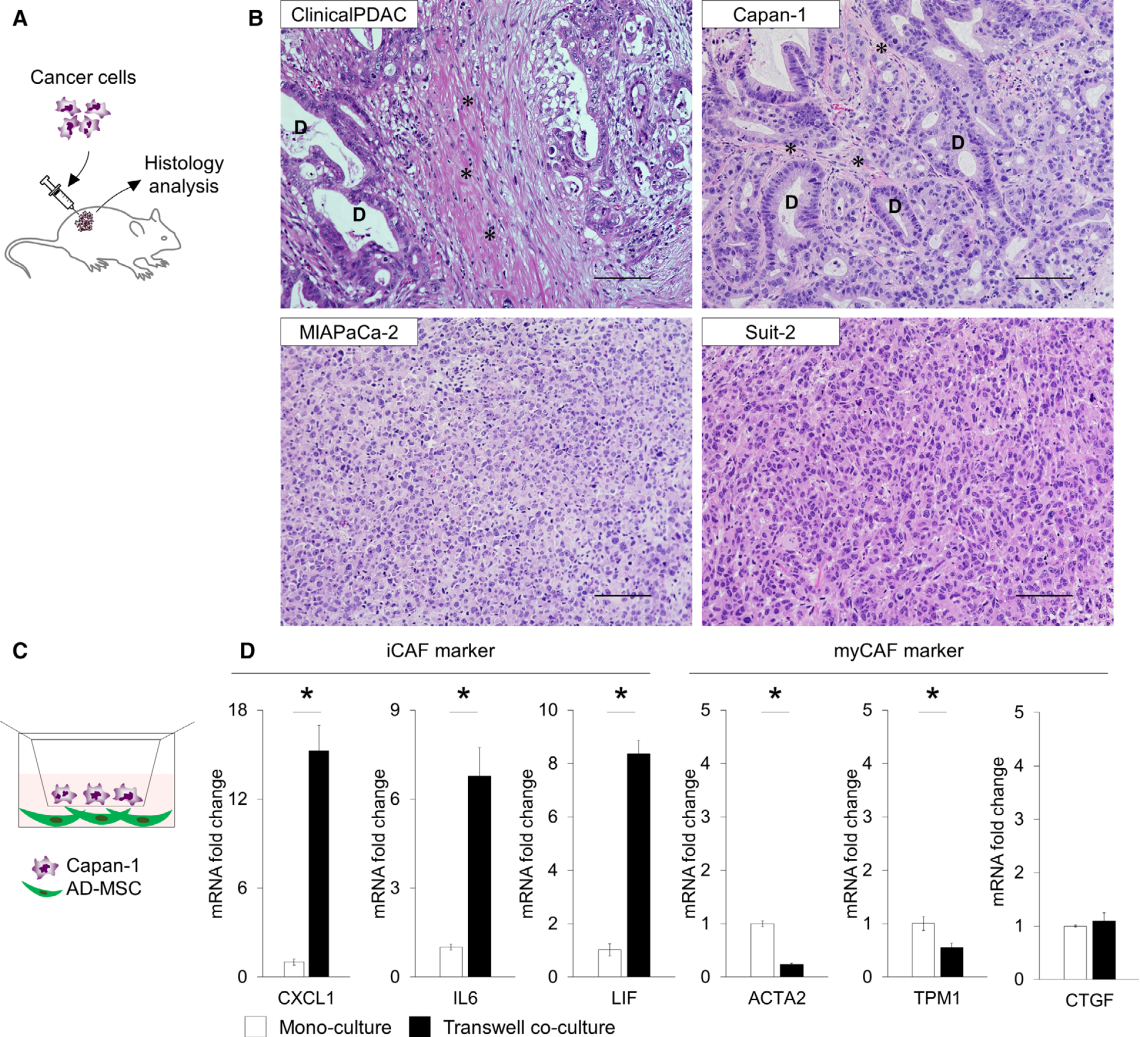
Co‐culturing AD‐MSCs with Capan‐1 cells using transwell‐induced differentiation into iCAF‐like cells. (A) Schematic illustration of the CDX model. (B) Histological analysis of HE‐stained human PDAC, and Capan‐1‐, MIAPaCa‐2‐, and Suit‐2‐derived xenograft. D, cancer duct structure. Asterisk indicates the stromal area. Scale bars: 100 µm. (C) Schematic illustration of the transwell co‐culture platform. (D) qPCR analysis of iCAF markers or myCAF markers in monoculture or transwell co‐culture. Results are presented as the mean ± SD of three biological replicates. *P < 0.05, unpaired Student’s *t*‐test.

We investigated which PDAC cell line effectively recapitulated clinical morphology, including aggressive tumor growth and CAF remodeling *in vivo*. First, we examined the histological features of the tumor in an *in vivo* xenograft mouse. Each cell line was cultured *in vitro*, after which the cells were transplanted into the dorsal subcutaneous space of immunodeficient mice to establish xenograft mice. After tumor formation, the tumors were harvested to evaluate their histological properties (Fig. 1A). The typical histological characteristics of PDAC include the presence of epithelial cancer cells forming ductal glands surrounded by abundant stromal components (Fig. 1B). In xenograft mice, Capan‐1‐derived cancerous tissue strongly exhibited organized gland formation and dense stromal proliferation. In contrast, MIAPaCa‐2‐ or Suit‐2‐derived cancerous tissue showed poor ductal glands and stromal area (Fig. 1B). Thus, Capan‐1‐derived cancerous tissue recapitulated clinical PDAC histology more accurately than the other cell lines. Therefore, we selected Capan‐1 as the representative PDAC cell line and conducted further *in vitro* experiments.

Next, we explored whether AD‐MSCs could be differentiated into CAFs *in vitro*. We co‐cultured AD‐MSC and Capan‐1 cells using the transwell system for 7 days (Fig. 1C); total RNA was extracted from the AD‐MSCs, followed by qPCR analysis. Thereafter, the expression levels of iCAF markers (*CXCL1*, *IL6*, *LIF*) and myCAF markers (*ACTA2*, *CTGF*, *TPM1*) were evaluated. Interestingly, in contrast to myCAF markers, iCAF markers were strongly upregulated in transwell co‐cultured AD‐MSCs (Fig. 1D). Of note, the expression level of the myCAF marker *ACTA2* was not upregulated compared to its corresponding expression level in the monocultured AD‐MSCs in all three PDAC cell lines (Fig. S1a,b). In contrast, the expression levels of the iCAF markers *IL6* and *LIF* were upregulated compared to their corresponding expression levels in the monocultured AD‐MSCs in all three PDAC cell lines (Fig. S1a,b). IF staining was performed after co‐culturing, although αSMA was not detectable in the AD‐MSCs (data not shown).

### Direct co‐culture with Capan‐1 cells induced the differentiation of AD‐MSCs into myCAFs

**Fig. 2:**
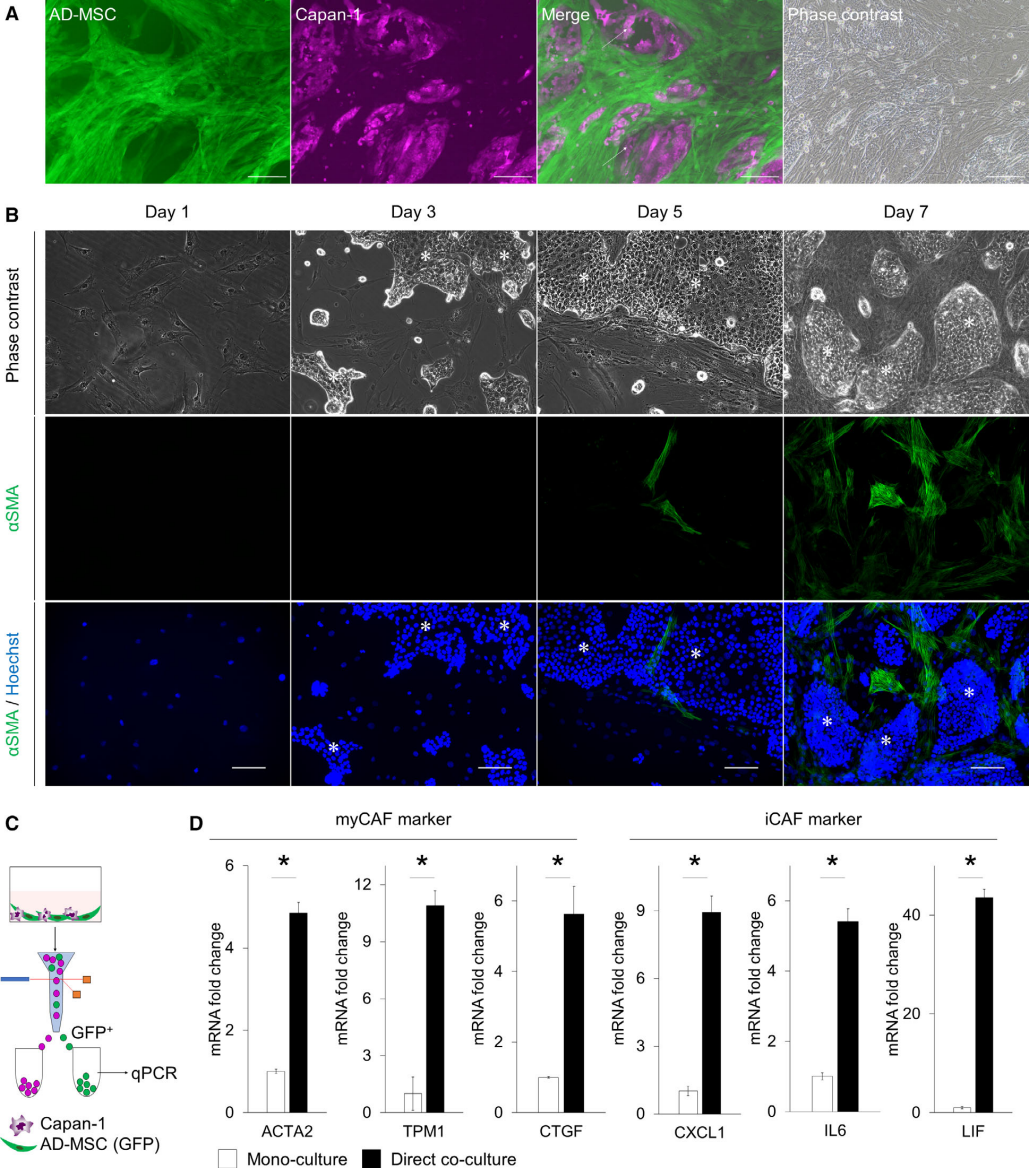
Co‐culturing AD‐MSCs directly with Capan‐1 induced differentiation into myCAF‐like cells. (A) Representative image of a direct co‐culture. Arrows indicate AD‐MSCs overlapped on Capan‐1s. Scale bar: 200 µm. (B) Representative IF image of AD‐MSCs directly co‐cultured with Capan‐1 stained for αSMA (green) on days 1, 3, 5, and 7. Counterstain: Hoechst 33342 (blue). Asterisks indicate Capan‐1 aggregations. Scale bar: 100 µm. (C) Schematic illustration of the direct co‐culture and flow‐cytometric sorting platform. (D) qPCR analysis of the myCAF marker or iCAF marker in monoculture or direct co‐culture. Results are presented as the mean ± SD of three biological replicates. *P < 0.05, unpaired Student’s *t*‐test.

Next, we investigated the role of cell–cell contact or short‐range paracrine signaling in CAF differentiation. AD‐MSCs labeled with GFP and Capan‐1 cells labeled with RFP were co‐cultured directly in common 2‐dimensional culture dishes. This enabled both cell lines to form 3D structures (overlapped in many layers or aggregations), with fringed spindle‐shaped MSCs surrounding Capan‐1 aggregations (Fig. 2A). Interestingly, the obtained morphology was histologically like that of clinical PDAC, in which abundant stromal cells are fringed around ducts formed by the cancer cells (Figs 1B and 2A). To investigate the role of cell–cell contact in CAF differentiation, IF staining in the direct co‐cultures was carried out. On day 5, αSMA‐positive AD‐MSCs were observed adjacent to Capan‐1 aggregations and their number was significantly higher on day 7 (Fig. 2B). Thus, we examined whether these AD‐MSCs had differentiated into myCAF. The GFP‐positive fraction was collected using FACS after 7 days of co‐culturing. Gene expression was analyzed by performing qPCR (Fig. 2C). As anticipated, the expression levels of myCAF marker genes (*ACTA2*, *CTGF*, and *TPM1*) were also upregulated, indicating that the direct co‐cultured AD‐MSCs were able to differentiate into myCAFs (Fig. 2D). On the other hand, the expression levels of iCAF marker genes (*CXCL1*, *IL6*, and *LIF*) were also significantly upregulated in co‐cultured MSCs, compared to those in monocultured AD‐MSCs (Fig. 2D), suggesting that direct co‐culture induced both iCAF and myCAF differentiation *in vitro*.

### AD‐MSCs showed dynamic morphological changes after direct co‐culture with Capan‐1 cells

**Fig. 3:**
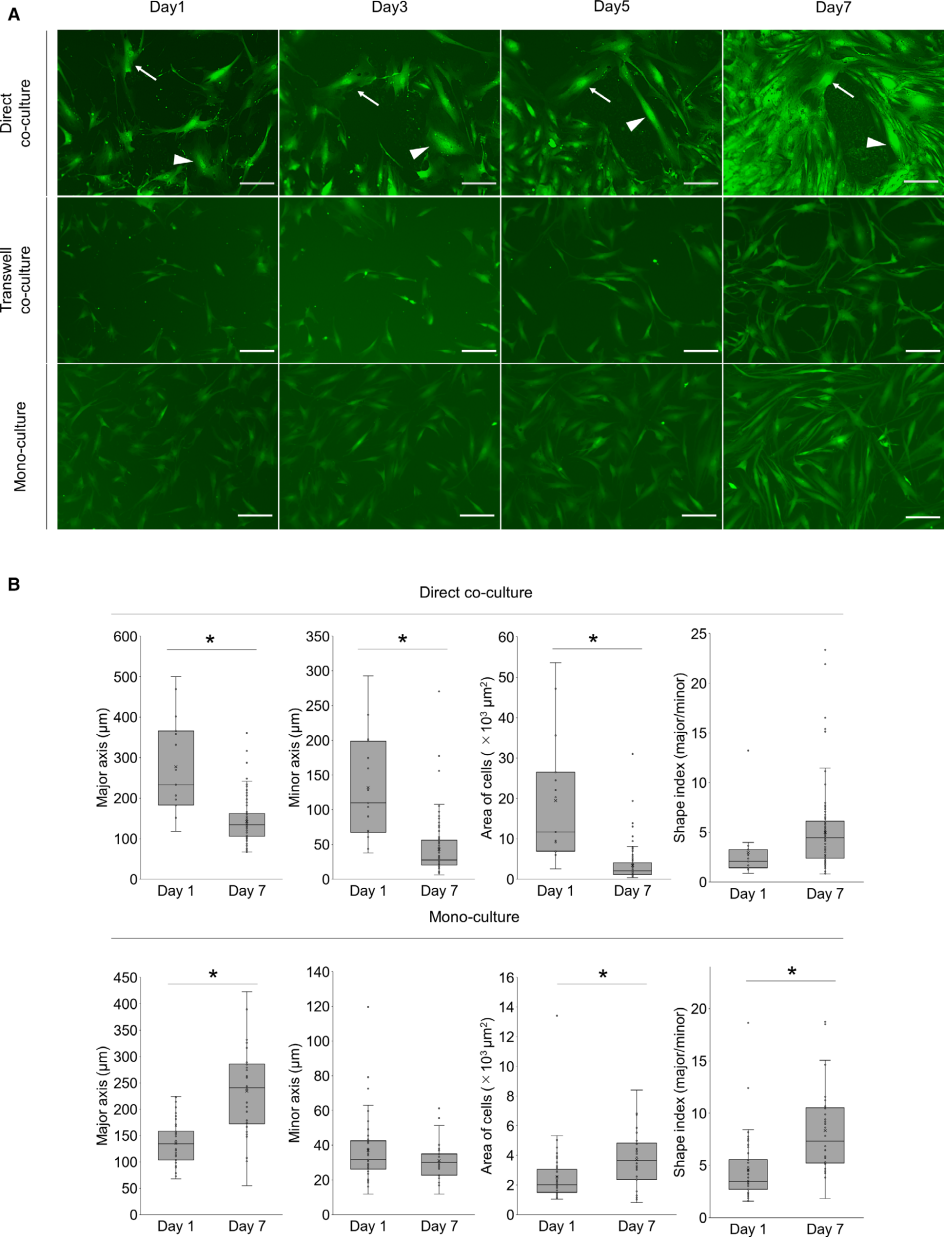
Co‐culturing AD‐MSCs with Capan‐1 induced differentiation into CAF‐like cells morphologically. (A) Representative time‐lapse IF images of monocultured or co‐cultured AD‐MSCs (green) with Capan‐1s. Arrows and arrow heads indicate AD‐MSCs in direct contact with Capan‐1. Scale bar: 200 µm. (B) The quantified values of AD‐MSCs in monoculture and direct co‐culture. Shape index indicates the ratio between the major and minor axis lengths. *P < 0.05, unpaired Student’s *t*‐test.

To examine the morphological alterations during the culture period, we performed time‐lapse recording of the direct and indirect co‐cultures (Fig. 3A). Of note, the morphology of AD‐MSCs did not vary during the culture period in both monoculture and transwell co‐cultures. However, in the case of direct co‐cultures, we observed various AD‐MSC morphologies, including large, small, spindle‐shaped, or stellate‐shaped. Particularly, we could observe large stellate‐shaped cells that were in direct contact with Capan‐1 cells. Soon after the contact occurred, their shape and size were dynamically changed (Fig. 3B). Specifically, the length of the major axis of the cell on day 7 became significantly shorter than that on day 1 in a direct co‐culture, while that in a monoculture became significantly longer. Moreover, the mean cell area became smaller in the direct co‐culture, whereas it became larger in the monoculture (Fig. 3B). On day 7 in direct co‐culture, the variation in the shape index, which indicates cell morphology was larger than that in monocultures.

### Global gene expression pattern indicated that AD‐MSCs could differentiate into CAFs *in vitro*

**Fig. 4:**
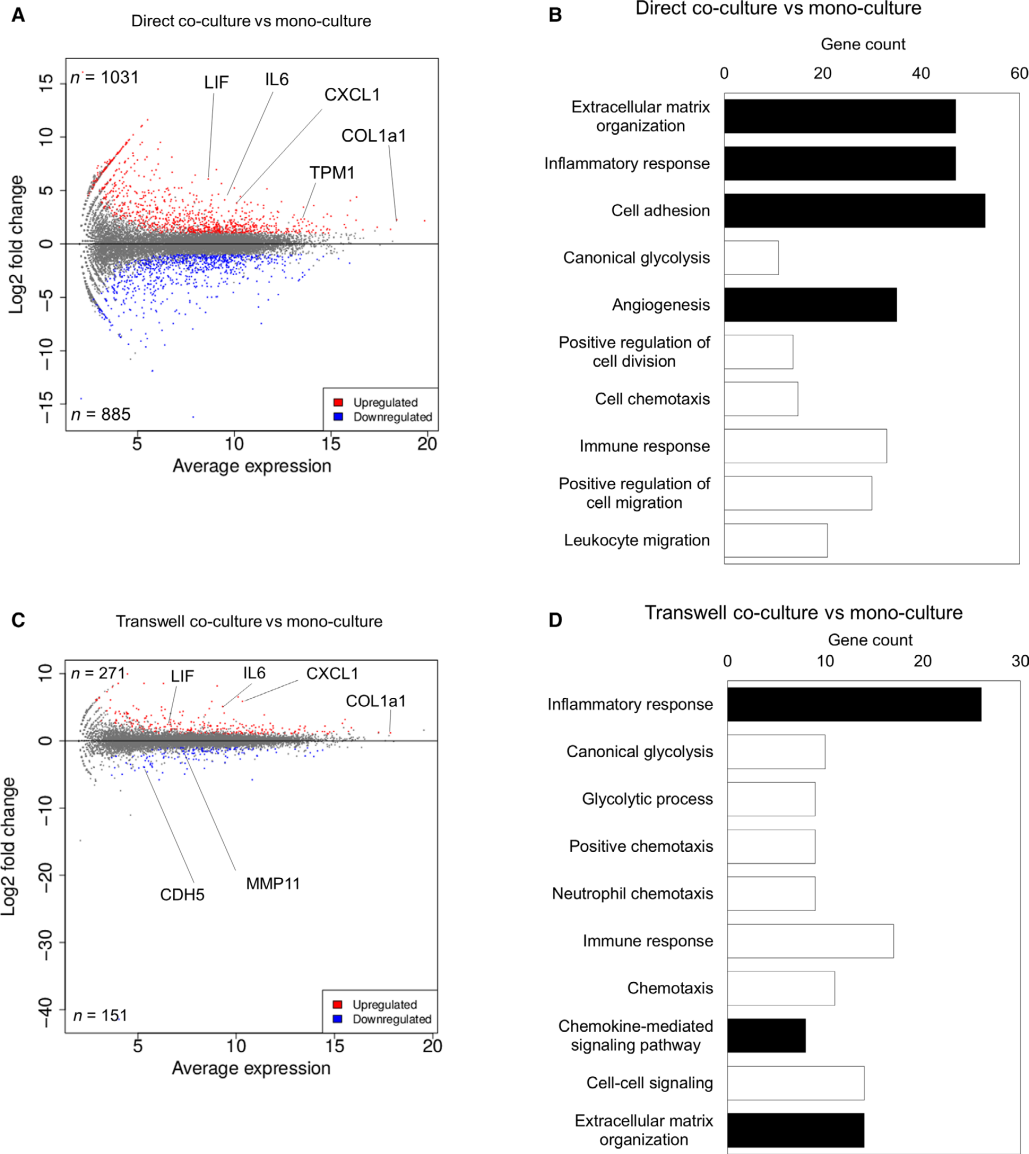
Global gene expression pattern showed that AD‐MSCs were differentiated into CAF‐like cells *in vitro*. (A) MA plot, a scatter plot of log_2_ fold change versus the average expression, showing differentially expressed genes (adjusted *P*‐value < 0.05 and log_2_ [fold change] ≥ 1) in a direct co‐cultured AD‐MSCs compared to those in monocultured AD‐MSCs. Upregulated genes are shown in red, and downregulated genes are shown in blue. (B) GO enrichment analysis of differentially expressed genes in direct co‐cultured AD‐MSCs compared to those in monocultured AD‐MSC. CAF‐related GO terms are shown in black. (C) MA plot, a scatter plot of log_2_ fold change versus the average expression, showing differentially expressed genes (adjusted *P*‐value < 0.05 and log_2_ [fold change] ≥ 1) in transwell co‐cultured AD‐MSCs compared to those in monocultured AD‐MSCs. Upregulated genes are shown in red, and downregulated genes are shown in blue. (D) GO enrichment analysis of differentially expressed genes in transwell co‐cultured AD‐MSCs compared to those in monocultured AD‐MSC. CAF‐related GO terms are shown in black.

We next performed a transcriptome analysis using RNA‐seq to compare the transcriptome profiles in differently cultured AD‐MSCs (transwell co‐cultured AD‐MSCs; direct co‐cultured AD‐MSCs) and clinical PDAC CAFs. We started by comparing the transcripts in direct co‐cultured AD‐MSCs with those in monocultured AD‐MSCs. Consequently, 1916 transcripts were differentially expressed, 1031 of which were upregulated (Fig. 4A). Among them, *IL6*, *LIF*, *CXCL1* (iCAF marker), *TPM1* (myCAF marker), and *COL1a1* (panCAF marker) were included. The upregulation of these myCAF markers and iCAF markers confirmed that AD‐MSCs were differentiated into both myCAFs and iCAFs in the direct co‐culture. However, *FAP* (a typical fibroblast marker) and antigen‐presenting CAF (apCAF) marker genes (*CD74*, *HLA‐DRA*) were not differentially expressed (Fig. 4A) [27]. Gene function was subsequently analyzed by performing GO enrichment analysis; genes that showed upregulated expression after direct co‐culture were significantly associated with GO terms such as ‘ECM organization’, ‘inflammatory response’, and ‘cell adhesion’ (Fig. 4B). Conversely, when the transcripts in transwell co‐cultured AD‐MSCs were compared with those in monocultured AD‐MSCs, 422 transcripts were differentially expressed (Fig. 4C). Among them, 271 transcripts were upregulated, including *IL6*, *LIF*, *CXCL1* (iCAF markers), and *COL1a1* (panCAF marker); however, myCAF marker genes (*ACTA2* and *TPM1*) were not included. The upregulation of iCAF markers confirmed that AD‐MSCs differentiated into iCAF in the transwell co‐culture. Similarly, *FAP* (typical fibroblast marker), *CD74*, and *HLA‐DRA* (apCAF markers) were not differentially expressed. Moreover, genes that had upregulated expression after transwell co‐culture were associated with GO terms such as ‘inflammatory response’, ‘chemokine‐mediated signaling pathway’, and ‘positive regulation of inflammatory response’ (Fig. 4D).

### Novel CAF markers were identified from transcriptome data of *in vitro* co‐culture model and clinical PDAC CAFs

**Fig. 5:**
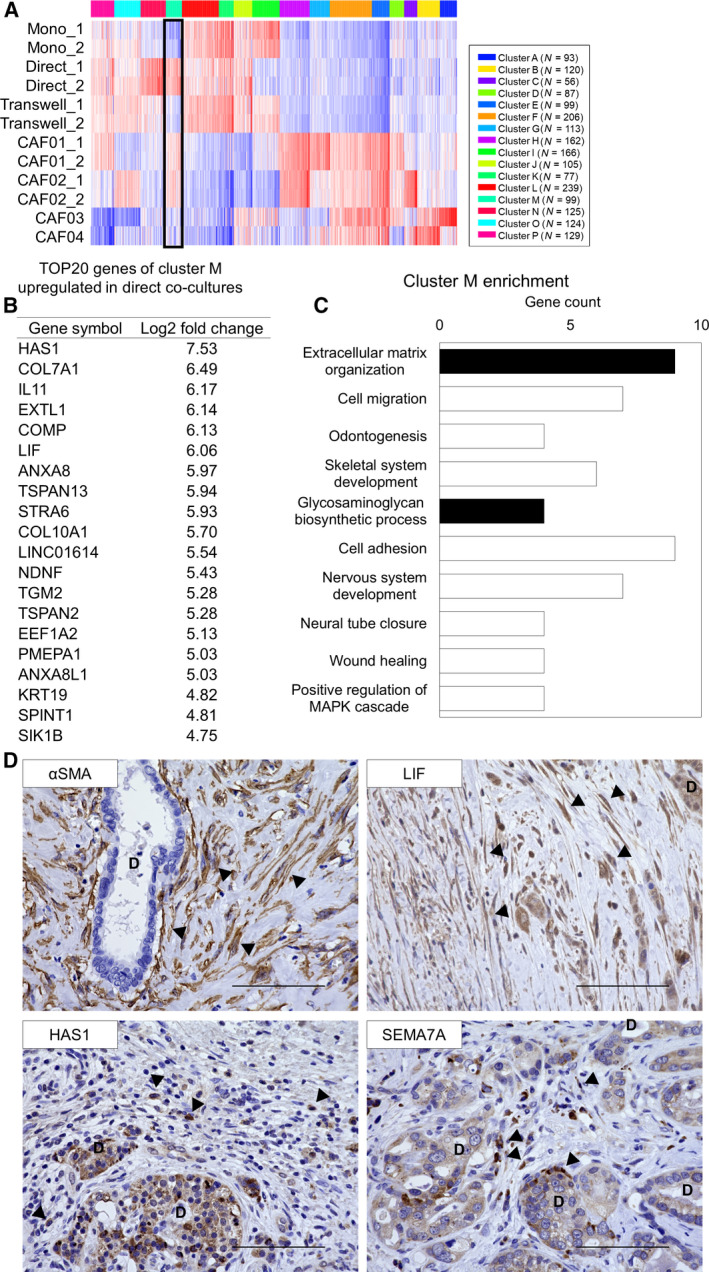
Potential CAF marker genes were identified by *k*‐means clustering of AD‐MSCs, co‐cultured AD‐MSCs, and clinical CAFs. (A) Heatmap of *k*‐means clustering. Cluster M (black square) included monocultured^low^, direct co‐cultured AD‐MSC^high^, clinical PDAC CAF^high^ genes. (B) List of the 20 genes in cluster M upregulated in a direct co‐cultured AD‐MSCs. (C) GO enrichment analysis of the aforementioned genes. ECM‐related GO terms are shown in black. (D) Representative immunohistochemical staining image of human PDAC for αSMA (brown, upper right), LIF (brown, upper left), SEMA7A (brown, lower right), and HAS1 (brown, lower right). Nuclei were stained with hematoxylin. Arrowheads indicate αSMA‐, LIF‐, SEMA7A‐, and HAS1‐positive CAFs, respectively. D, cancer duct structure. Scale bar: 100 µm.

Finally, we implemented a hierarchical clustering to identify clear differences between the *in vitro* co‐culture model and clinical PDAC CAFs (Fig. 5A). One cluster of the *k*‐means heatmap (Cluster M in Fig. 5A) was characterized as monocultured AD‐MSC^low^, direct co‐cultured AD‐MSC^high^, and clinical PDAC CAF^high^. This cluster contained 99 transcripts, including the myCAF and iCAF genes, such as *ACTA2* and *LIF*, as well as additional genes including *HAS1*, *IL11*, *DKK2*, and *SEMA7A* (Fig. 5B, Table S1). GO enrichment analysis using these genes confirmed that they are associated with ECM‐related GO terms (Fig. 5C), indicating that the AD‐MSCs have acquired the function of ECM remodeling, which is a core CAF characteristic [28, 29]. To confirm whether the enriched genes existed in clinical PDAC, we performed IHC staining of clinical PDAC using the positive markers, αSMA and LIF, as well as the novel potential markers, HAS1 and SEMA7A (Fig. 5D). *HAS1* was previously reported as one of the expressed genes in the iCAF, whereas the expression pattern was not examined previously. *SEMA7A* has not been reported in PDAC. The αSMA‐positive myCAFs were located adjacent to the cancer duct structure and at distant places (Fig. 5D upper right), whereas the LIF‐positive iCAFs were located mainly far from the cancer cells, as previously reported (Fig. 5D, upper left). On the other hand, HAS1‐positive fibroblasts were distant from cancer cells, as expected. Interestingly, SEMA7A‐positive fibroblasts were located only in the restricted area that was close to the cancer cells. Of note, cancer cells were also positive for LIF, SEMA7A, and HAS1, while they were negative for αSMA.

## Discussion

In this study, the *in vitro* co‐culture system for improved simulation of CAFs differentiation from AD‐MSCs was conclusively established. As anticipated, AD‐MSCs were differentiated into distinct CAF subpopulations when employing two different co‐culture systems *in vitro*. In addition, we used omics data to identify potential CAF markers that would aid in investigating the mechanisms underlying the role of CAFs in the TME.

Recent studies have suggested that CAFs were derived from MSCs, which may be either populated in the pancreas or recruited by neoplastic cells [7, 30]. Whereas pancreatic stellate cells from mice or from human patients were used in previous studies [31, 32], they were unstable and quite variable, as their characteristics were not accurately assessed. We focused on AD‐MSCs as the source of CAFs because they are available, easy to handle, and are multipotent, allowing their differentiation into CAFs [33, 34]. In fact, several published reports revealed that AD‐MSCs differentiated into CAFs [35, 36]. However, it has not been confirmed whether AD‐MSCs would differentiate into heterogeneous CAFs. Based on these findings, an immortalized AD‐MSC cell line, ASC52telo, was selected for this study.

In CAF differentiation, we considered direct cell–cell contact between cancer cells and CAFs as a key factor. Direct cell–cell signaling, such as programmed cell death 1/programmed cell death ligand 1 signaling, or mechanical stress between cancer cells and other surrounding cells is important in the TME. In addition, cancer cells and CAFs communicate with each other *in vivo*. Therefore, to recapitulate the TME *in vitro*, we employed co‐culturing using the transwell system as an indirect co‐culture. It was confirmed that AD‐MSCs were differentiated into iCAFs in the transwell co‐culture; conversely, they were differentiated into myCAFs in a direct co‐culture, as demonstrated in qPCR analysis. The mRNA expression levels in AD‐MSCs varied according to different culture conditions (Figs 1D and 2D), indicating that AD‐MSCs could differentiate into iCAFs when in co‐culture with Capan‐1 cells using a transwell system. This type of a culture system allows paracrine interactions but prevents direct cell–cell contact, which was needed to induce myCAFs. In addition, using time‐lapse imaging and chronological IF staining during co‐culture, it was revealed that direct cell–cell contact was a key factor in the differentiation of CAFs, both transcriptionally and functionally, especially for myCAF differentiation (Figs 2B and 3A). The cell morphology began to change at the beginning of the co‐cultures, but αSMA expression only began at around day 5; this implied that the morphological changes may initiate αSMA expression and CAF differentiation.

RNA‐seq and GO enrichment analysis of the selected gene cluster demonstrated that direct co‐culture induced AD‐MSCs into more physiologically relevant CAFs. Additionally, by focusing on the similarity to clinical CAFs, we could identify potential CAF markers (Fig. 5B, Table S1). These included not only previously identified CAF genes but also the unrecognized genes related to PDAC [27, 29]. We performed IHC staining for the representative potential markers in clinical PDAC, confirming their expressions in CAFs (Fig. 5D). Therefore, the gene cluster analysis may be useful in identifying unrecognized markers including novel medical seeds for targeting CAFs and TME in PDAC.

Although this *in vitro* study has benefits in pharmacological manipulation, genetic modifications, and imaging analysis, our study also has several limitations. For example, the *in vitro* co‐culture system is a restrictive environment compared to an *in vivo* model. This could have been a reason that we identified apCAF markers that were not upregulated in the co‐culture model. Originally, the apCAF population was discovered by the analysis of GEMM and clinical PDAC samples [27]. This indicates that the differentiation into apCAFs can be correlated with the immune response mechanisms. Thus, the *in vitro* co‐culture model is a constrained environment; however, 3D co‐culture models, or co‐culturing with multiple cell types, could expand the available possibilities.

In summary, we confirmed that AD‐MSCs could be differentiated into distinct CAF subtypes depending on the different co‐culture conditions *in vitro*, and our co‐culture system would be useful for investigating the functions of CAF, which combines AD‐MSCs and Capan‐1 in effectively reconstructing a pancreatic TME like that of clinical PDAC. Although several functions of CAFs have been elucidated in recent studies [37, 38], numerous variables remain unclear, which calls for more comprehensive studies. Prospectively, we plan on investigating selected potential CAF marker genes that were identified in our omics data to clarify their relationship with CAF functions and cancer progression.

## Conclusions

AD‐MSCs can differentiate into distinct CAF subtypes depending on the different co‐culture conditions *in vitro*, and we identified potential CAF markers that would aid in investigating the mechanisms underlying the role of CAFs in the TME.

## Conflict of interest

The authors declare no conflict of interest.

## Author contributions

YM and YSK conceived the study design. YM performed the experiments. YM, NM, and YSK analyzed the data. TO, and YSK supervised the experimental implementation. YM and YSK wrote the manuscript. All authors reviewed the manuscript and approved the final version for submission.

## Supporting information


**Fig. S1.** Capan‐1 induced cancer‐associated fibroblast (CAF) differentiation to the maximum extent among pancreatic cancer cell lines. (a) Schematic illustration of the transwell co‐culture platform. (b) qPCR analysis of representative CAF markers in mono‐culture or transwell co‐culture. Results show the mean ± SD of three biological replicates. *P < 0.05.

**Table S1.** Gene list of cluster M in *k*‐means clustering.

## Data Availability

RNA‐seq data are available in the DDBJ Sequence Read Archive under accession number DRA010287. The raw data are available from the corresponding author upon reasonable request.
