# Supplementary material for: Adipose‐derived mesenchymal stem cells differentiate into pancreatic cancer‐associated fibroblasts in vitro
Source: FEBS Open Bio. 2020 Oct 9;10(11):2268–81. doi: 10.1002/2211-5463.12976 (PMC7609785; doi:10.1002/2211-5463.12976)
Supplement: Supplementary file 1 — Fig. S1. Capan‐1 induced cancer‐associated fibroblast (CAF) differentiation to the maximum extent among pancreatic cancer cell lines. (a) Schematic illustration of the transwell co‐culture platform. (b) qPCR analysis of representative CAF markers in mono‐culture or transwell co‐culture. Results show the mean ± SD of three biological replicates. *P < 0.05. [file FEB4-10-2268-s001.docx]

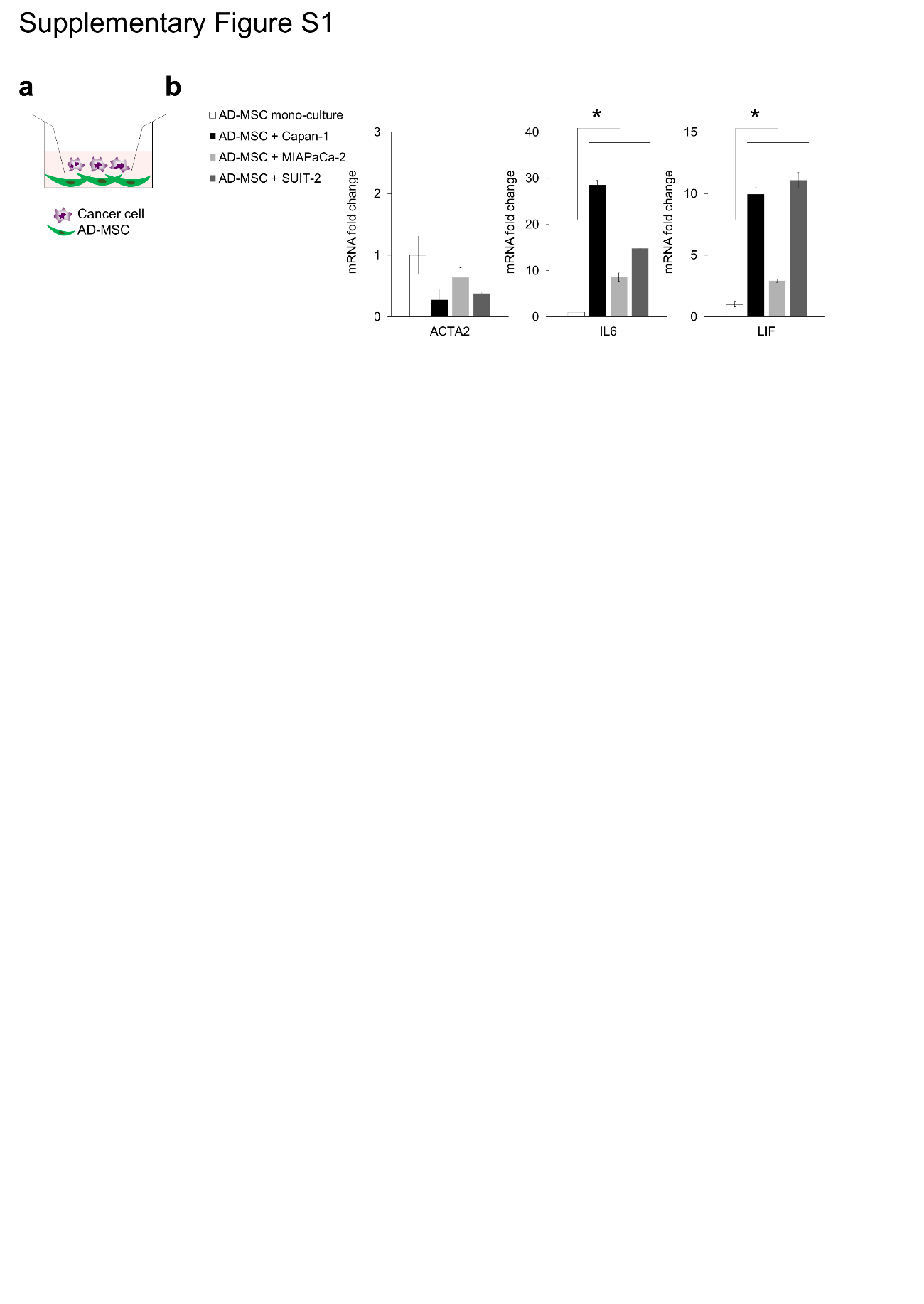


**Supplementary Figure 1. Capan-1 induced cancer-associated fibroblast (CAF) differentiation to the maximum extent among pancreatic cancer cell lines.**

(a) Schematic illustration of the transwell co-culture platform.

(b) qPCR analysis of representative CAF markers in mono-culture or transwell co-culture. Results show the mean ± SD of three biological replicates. *, P < 0.05.
